# Psychotic-like experiences among university female students in Qatar: A qualitative-phenomenological study

**DOI:** 10.3389/fpsyt.2022.988913

**Published:** 2022-09-23

**Authors:** Arij Yehya, Salma M. Khaled, Iris E. C. Sommer, Peter Woodruff, Suhad Daher-Nashif

**Affiliations:** ^1^Core Curriculum Program, Qatar University, Doha, Qatar; ^2^Social and Economic Survey Research Institute, Qatar University, Doha, Qatar; ^3^Department of Neuroscience and Department of Psychiatry, University Medical Center Groningen, Groningen, Netherlands; ^4^Psychiatry Department, Hamad Medical Corporation, Doha, Qatar; ^5^Department of Neuroscience, School of Medicine, University of Sheffield, Sheffield, United Kingdom; ^6^Department of Population Medicine, College of Medicine, Qatar University (QU) Health, Qatar University, Doha, Qatar

**Keywords:** psychotic-like experiences, non-clinical sample, university students, culture, religion, Middle East

## Abstract

**Background and aims:**

Psychotic-like experiences (PLEs) are hallucinatory or delusional experiences that fall below the threshold of a diagnosable psychotic disorder. Although PLEs are common across the spectrum of psychiatric disorders, they also have been commonly reported in the general population. In this study, we aimed to describe the types of PLEs experienced by university students in Qatar. Furthermore, we aimed to examine how students frame, explain, and deal with these experiences as well as understand how culture and religion may shape the way students attribute and respond to these experiences.

**Method:**

This study used a qualitative phenomenological approach. For collecting the data, we conducted semi-structured interviews using the Questionnaire for Psychotic Experiences (QPE). The QPE is a valid and reliable tool to assess the phenomenology of psychotic-like experiences. The questionnaire was translated into Arabic and tested and validated in Qatar (a fast-developing Muslim country in the Arabian Peninsula). We conducted interviews in Arabic with 12 undergraduate female students at Qatar University (the only national university in Qatar). The interviewees were of different Arab nationalities. Interviews were transcribed verbatim and two authors conducted the content-thematic analysis separately, as a strategy to validate the findings. The study was part of a larger nationally funded project that was approved by the Qatar University Institutional Review Board. The approvals were granted before any interview was conducted.

**Results:**

The PLEs were prevalent in our non-clinical sample. The content-thematic analysis revealed the following main themes about these experiences: type, impact on daily function, frequency, immediate reaction, attribution style, assumptions about the root cause of these experiences, other associations, and religious links to experiences. The results also highlighted that religion and culture play a role in shaping the types of hallucinations and some delusions.

**Conclusion:**

Our findings support the importance of culture and religion in relation to the types and explanations that students provided when describing PLEs. Notably, it was common among those who reported having these experiences to normalize and link PLEs to real-life events. This may be a defense mechanism to protect the self against the stigma of mental illness and from being labeled as “abnormal”.

## Introduction

Auditory and visual hallucinations and delusions are commonly associated with psychotic disorders according to the Diagnostic and Statistical Manual of Mental Disorders (1). People who were diagnosed with psychotic disorders, for example, believed that others were poisoning their food. These experiences impact their daily functioning, so they are unable to carry out daily tasks and activities such as leaving the house and watching TV (2).

Psychotic-like experiences (PLEs) are prevalent among the general population (3, 4). The prevalence varies across cultures depending on the assessment instruments and the population studied (5). Learning more about these experiences in non-clinical samples of young adults can provide further information on schizophrenia-related risks, including cognitive, environmental, and linguistic factors (6). Non-clinical samples also present with depressive symptoms due to PLEs (7).

The new and psychometrically validated questionnaire, known as the Questionnaire for Psychotic-like Experiences (QPE), is suitable for use in non-clinical samples (8). The QPE provides insights into the prevalence and phenomenology of PLEs such as distress, severity, and their impact on daily functions.

In the general population of Qatar, Khaled et al. (9) showed that the prevalence of PLEs was 27.9%, with visual hallucinations being the most common in this sample. In addition, Arabs had a significantly higher prevalence of PLEs than non-Arabs. Consequently, in the Arab sample, women had a higher prevalence of PLEs than men, with psychological distress being a common feature of PLEs in this sample. Therefore, it is vital to learn more about these experiences among women Arabs and to understand the attributional style, as well as to learn the source of the distress caused by these experiences.

In a sample of patients with psychosis in the United Kingdom (UK), Hirschfeld et al. (2) showed that themes such as personal explanation, personal/interpersonal changes, and immediate expression of psychotic experience were common. Moreover, patients reported either avoidant or expressive coping strategies. They also reported a struggle in maintaining a sense of self due to psychosis. In these cases, patients tend to isolate themselves and experience distress due to these interpersonal changes.

Thus, it is important to learn whether these experiences are similar among non-clinical samples. Brett et al. (10) showed that distress was reduced in non-clinical samples in which psychotic experiences were normalized and accepted by the social context around the individual. Heriot-Maitland et al. (11) even suggested “normalizing” PLEs as a potential therapeutic strategy for clinical patients.

The QPE was translated to Arabic and validated in both clinical and non-clinical samples of Qatar. However, when administered quantitatively in large samples, our ability to understand these lived experiences, stories, and the essence of these psychotic-like delusions and hallucinations from the point of view of the person themselves is hindered. Understanding these experiences phenomenologically enables us to compare them with patients with psychosis (12).

Past studies reported on culture as a sociocultural factor that shapes hallucinations (13, 14). Culture has an impact on the frequency, content, and reaction to psychotic experiences (13). For instance, the frequency of students experiencing PLEs was higher in Nigeria than in Dutch or Norwegian samples (14). Regionally, a study from Saudi Arabia conducted a thematic analysis of data representing the psychotic experiences of 59 individuals and found that Saudis report religious (e.g., Jinns) and cultural (e.g., modest clothing) frameworks alongside biological, psychological, and social mechanisms that potentially trigger an alternative reality for the affected individual (15).

In this study, the aim was to describe the phenomenology of PLEs reported by female university students in Qatar. In addition, the aim was to examine how students view, explain, and deal with these experiences. In turn, we also wanted to understand how culture and religion shape the way students frame and respond to these experiences. For these purposes, we built on the QPE questionnaire by asking in-depth open-ended probes, in addition to the main questions of the questionnaire. This allowed us to learn more about the experiences and understand how the students refer to these experiences and explain them in their own words. It should be noted that this study is the first study of its kind in Qatar and among very few conducted in the Arab region on this subject matter. Qatar is a culturally diverse country, with almost 100 nationalities from all over the globe. It is considered one of the richest and fastest developing countries in the Arabian Peninsula.

## Methods

The current study was part of a nationally funded larger study aimed at estimating the prevalence and describing the phenomenology of psychotic experiences in Qatar. More information about the study is available at http://pe-qatar.com/. Qatar University Institutional Review Board approved the study (QU-IRB 1021-EA/19) before any data collection.

In this part of the study, we used a qualitative phenomenological approach to understand the PLEs of female university students. We chose the qualitative phenomenological approach because it enables the researcher(s) to examine the structure and essence of the participants' lived experiences and to investigate the everyday experiences of human beings while suspending the researchers' preconceived assumptions about the phenomenon (16, 17). The study included 12 Arab female students of different nationalities. An advert was sent out by email to students. Those who were interested to participate in the study were asked to reach out to the researcher *via* email or phone. After that, a virtual session was scheduled. The inclusion criteria were as follows: participants must be enrolled as full-time students at Qatar University at the time of the study and must be at least 18 years old. These criteria were mentioned in the call to participate. Hence, all participants who were interested in the study met the inclusion criteria, and no one was excluded from the study.

Informed consent was obtained verbally on record before the beginning of the interview. This way of seeking consent was approved by the IRB committee. All participants were informed about their right to decline participation altogether, can withdraw from the study at any time, and can refuse the audio recordings of interviews. For confidentiality purposes, no participants' identification data (such as name, age, etc.) were used in any of this study's outputs.

To collect the data, we used semi-structured interviews based on the QPE. The full interview guide is available as a Supplementary Appendix. Participants were asked open-ended questions to probe about these experiences in detail. In addition, given that the QPE includes many types (e.g., auditory, visual, tactile, and olfactory) of psychotic experiences, the interview was conducted over three rounds to make sure that the transcriptions of the interviews covered the entire questionnaire. While we asked about each of the hallucination types and their impact on the individual, we grouped the delusions together by asking about their impact only once. This was to make more time within the interview to elaborate on the experience itself. Interviews were conducted in Arabic using the online video-conferencing platform Microsoft Team due to the COVID-19 pandemic. Interviews took between 45 and 60 min to complete. The online interview was scheduled based on the preferred time suggested by the participant. The participants had the choice to have their camera switched on or off during the interview, meanwhile the interviewer had to have the camera on throughout the interview. During the interview, the main questions that are in the QPE questionnaire were shared on screen. Nevertheless, the interviewer read these questions and followed them up with probing to learn more about the experiences in more detail. The interviews were recorded *via* Microsoft Teams, and after that, all interviews were transcribed verbatim. Relevant quotations were translated to English and then translated back to Arabic to ensure the validity of the translation.

For analyzing the data, we used the inductive analysis of the participant's responses to each of the hallucinations (auditory, visual, tactile, olfactory, and multimodal) and the delusions (paranoid, reference, guilt, control, religiosity, and grandeur). This enabled us to identify patterns within the data to descriptively report each one of these experiences.

Two Arabic-speaking researchers with a background in psychology and sociology-anthropology analyzed the first two interviews separately, confirmed the main themes of analysis, and continued to analyze the remaining interviews separately. The analysis began with open coding (for identification), followed by axial coding (for comparison), and lastly with descriptive themes that we present in the next section.

Twelve students met the inclusion criteria. All students completed the interview. Participants were aged 19–24 years, with all of them being single women (Table 1). Although the call to participate in the study targeted men and women equally, only female students responded and expressed interest in participating in the study. This might be explained by the results of Khaled et al. (9), who found that, in the general population of Qatar, Arab women had a higher prevalence of PLEs as compared to Arab men. This can also be explained by the social–cultural stigma surrounding mental health, which frames mental illness as a weakness that negatively impacts men's masculinity. We did not ask male students directly to participate to maintain the voluntary participation component and to avoid any impact on hierarchal relationships between the students and us as faculty.

**Table 1:** Participants' characteristics.

**Participant #**	**Age**	**Gender**	**Region**	**Field of studies**	**Marital status**
1	18–20	Female	Arabian Peninsula	Social Sciences	Single
2	21–25	Female	Northern Africa	Humanities	Single
3	21–25	Female	Levant	Behavioral Sciences	Single
4	21–25	Female	Arabian Peninsula	Humanities	Single
5	21–25	Female	Northern Africa	Behavioral Sciences	Single
6	18–20	Female	Northern Africa	Life Sciences	Single
7	21–25	Female	Levant	Environmental Sciences	Single
8	21–25	Female	Arabian Peninsula	Humanities	Single
9	21–25	Female	Arabian Peninsula	Humanities	Single
10	18–20	Female	Levant	Social Sciences	Single
11	18–20	Female	Levant	Humanities	Single
12	18–20	Female	Levant	Environmental Sciences	Single

## Results

The analysis revealed nine themes for each type of hallucination and delusion. These themes are as follows: type, impact on daily function, frequency, immediate reaction, attributional style, assumptions about the root cause of these experiences, other associations, and religious links to experiences. In this section, we will present each one of these nine themes in each hallucination and delusional experience.

### Auditory hallucinations

Seven students reported experiencing one or more auditory hallucinations. The content of these experiences included mainly three types of voices/sounds: a call for prayer (*Azan*), a phone/mobile ring, and/or a call from a family member, mainly a mother or a sister. Only one participant mentioned negative content related to the voices being heard, but the majority of the participants did not report negative content. For instance, participant 3 said,

Sometimes I hear the voice of Azan but when I open the window to assure what I heard, I don't find a voice…rarely I hear my mother calls me but when I close the shower I don't find any one calls me.

Similarly, participant 6 said,

I think I heard the voice of the Azan, I said to a person near me if there's a call for prayer and he said no, there is no Azan yet.

The majority of the participants pointed out that they heard these voices one time a month, but few (two participants) heard the voices one time a week. The main and immediate reaction shared by the participants was to check whether the sound/voice was there or existed. If they heard the voice while they were showering, they closed the shower and checked or asked the person who they heard calling them. They opened windows to make sure of the voice they heard or asked somebody close to them. For example, participant 2 said,

When I hear somebody calling me, I close the water and open the door to ask if somebody called me…they say no, and then I am sure no one called and I close the door

Most participants framed these voices as inner voices and illusions coming from inside their heads. Despite framing them as inner voices or illusions, they believed that they might be real. Only one student framed them as unreal voices, whereas one framed them as absolutely real. The students tried to normalize the voices/sounds by referring to them as normal experiences that many people they know experience. They reported strange feelings when they experienced these voices for the first time, but with time, they adapted to it. The assumed reasons for these experiences as suggested by the students were mainly life events. For instance, participant 1 said:

There was a period where the home phone would not stop ringing…my friends continuously called me so the I continued to hear voice of the ringing phone continued even when the calls stopped […] we are moving home and the fear of doing the wrong thing sometimes make negative inner voices […] also many bad things happened in my home country Yemen and the war there […]

Participant 8 also mentioned how life events such as a trauma with a friend were the reason she kept hearing her friend's voice and said,

I heard my friend calling me. When I was in grade two I think I have a trauma in my mind…She suffered from family problems…her father used to beat her…so I would hear her calling me

One participant related the voices to biological reasons and said “It's our brain.” Another one linked the voices to “*Jinn”*.

When the students were asked about what came to their mind when we asked about negative voices/sounds, the majority of them associated the question with social critique regarding their body, behavior, and academic performance. This indicates another attempt to normalize voices by linking them to normal real-life experiences. As part of linking the voices to reality, the students linked their auditory experiences to religion mainly by attributing them to Satan. For example, participant 5 stated

I am a person who is generally very afraid so the first thing I think about is Jinn and these things…To answer the question on whether or not these voices are real, yes I believe they are real but their resources are spirits or Jinn.

Participant 8 also disclosed that,

Personally I suffer from obsessive compulsive disorder that is related to things like washing and cleaning my hands. I don't think I am the source of these things and I feel their source is unknown...i don't know...is it Satan? I honestly don't know.

Attributing their experiences to religious themes was a common practice among these students. This can be referred to as a strong influence of religion on their daily lives as well as being a huge part of their belief system and cultural norms.

### Visual hallucinations

Out of 12 students, five reported experiencing visual hallucinations. The subject matter of these experiences was mainly moving shapes or shadows that emerged from static real objects. For example, participant 4 said,

The only weird objects I've seen are =things moving fast, it's either white or black. Sometimes I see a face or animal shapes on carpets or ceramic surfaces or trees…

Similarly, participant 7 said,

[…] for example when there's Abaya hung in front of me…spontaneously my brain translate it that there's somebody there… I feel uncomfortable, so I change its place or remove it. Sometimes I see shades […], like sometimes while I'm sitting I look on the floor …our floor is made of marble with many lines like veins…when I stare at the pattern, I sometimes see a shape of face or a picture that I saw on Instagram or Twitter…it's a pattern in my head…it's for a few seconds and then it disappears.

The above participant was one of the two that mentioned that they experienced a minor discomfort while experiencing these hallucinations. The others said that it did not affect them in any way. Their answers failed to indicate a consistent pattern of frequency, but there were different frequencies reported by different participants and it ranged from once a day to once a year. Furthermore, all participants stated that they do not interact with these objects, rather they react to confirm their presence. An example of this is highlighted in the above quotation from participant 7. Another example is a statement made by participant 4:

I look and look on the surface area where I saw this weird object…I sit steadily…look…search…and check if there is really an object…if there is a weird object or these are only illusions.

Despite framing these experiences as illusions by the majority of those who experienced visual hallucinations, they thought that they might be real, but not 100% real. Only one participant (Participant 1) framed these experiences as resulting from a disease and said, “I feel those are the result of an existing disease …I see on YouTube they say the panic attack is when the person cannot move and can hear voices and imagines things”.

Four participants framed these experiences as normal as almost everyone experienced and experiences them.

Participant 5 said, “Personally, I think almost all of us passed through such experiences”. Normalizing these experiences and referring to them as partially real can be linked to the notion that participants linked these experiences to religion. For instance, participant 2 asserted:

The issue could be linked to culture and religion…for example there are fixed things in religion, such as the existence of Jinn and the existence of the other world existence…these things are fixed. So the idea that we ignore them does not mean we oppose to religious text.

Participant 7 added,

I remember the reaction of people to these things; some say for example that this might be Jinn, which might be real according to Muslims. Some may this is not jinn rather it is a real creature

The reasons given for having these experiences can be divided into four categories: past experiences especially watching horror movies or traumatic life events, mental and psychological issues, such as depression, and one person assumed that physical environmental features such as light and shadows make these shapes.

Participant 1, for example, said,

I think people who watch many horror movies they see many like this…I know my friends who watch these movies frequently have such experiences where they see hear things or see things moving. They always tell me they hear voices and see Jinn and so on. …

When asked about associations with the question, participants remembered their own experiences. However, those who did not experience visual hallucinations remembered the experiences of their friends or relatives who told them about these experiences. Participant 4, for example, remembered what her sister had told her and said,

I thought of people who see strange objects in front of them…for example my sister has experienced this. She was walking and suddenly a weird creature appeared in front of her face. The weird thing is that the creature stayed still for a period of time until she repelled.

Remembering stories told by friends and relatives, then linking these experiences to religion, and reporting on the lack of interaction or impact support, highlights how the participants continuously attempt to normalize and find logical explanations for these experiences, even if they are not the ones who personally experienced them.

### Olfactory hallucinations

Four participants reported that they experienced olfactory hallucinations in the past, while two said maybe yes but they are not sure. The content of these experiences mainly revolved around perfume, cigarettes smoke, fire smoke, or the smell of certain types of food. Participant 2, for example, said,

Sometimes while I'm sitting I smell a beautiful perfume that I had smelled in the past. But there's no one there in the room except me and it is not the smell of the perfume I am wearing, so the source of the smell remains unknown.

Participant 7 reported,

My father is a smoker…even when he's not at home or he's not in the same place with us… I can smell the cigarettes smoke… but after a while we stop smelling it. It's weird. Sometimes we think even when we are having a meal we taste and smell it.

Participants reported these experiences as rare occurrences. Their main reaction is to look for the main source of these experiences, but if they do not find it, they tend to seek refuge in religion and God. As participant 6 said, “when it's not there I seek refuge in Allah and the smell disappears…I don't know where it goes”. Seeking refuge from Satan was mentioned by one participant as a recommendation from her mother when she has such experiences. She said,

When I smell the food or something burning. I look around and don't find the source of the smell, my mother says “this is from Satan, seek refuge in Allah and the smell will fade.”

Participants framed these experiences as illusions that many people experience. These attempts to normalize such experiences were present when they tried to explain the reasons behind them. According to participants, the reasons behind these experiences are past experiences or spiritual notions namely related to Jinn.

Two participants said that these experiences are the results of doings by Jinn or by Satan. However, others said traumas. For example, participant 8 mentioned,

The reason is trauma. For example when a person's home has been burned down…he vividly remembers the smell the fire smoke and his fear for his family or maybe he even lost a family member…So after years when he can suddenly smell smoke and he directly he starts having a panic attack although there's no smoke but previous traumas may have triggered this

Two participants asserted that it is all linked to our memory. What we smell is related to what we have smelled in the past, and with certain triggers, it all comes back to memory *via* certain mechanisms inside the human brain.

### Tactile hallucinations

Four participants reported experiencing tactile hallucinations. The subject of these experiences includes two types: feeling a hand on one's shoulder and something “walks” on/in/through the body. For example, participant 11 talked about in or on the body tactile hallucinations and said,

I feel something running in my body. It's like a specific sensation. A small body runs…follows me…touches me either a hand or like something tickling me and making me feel itchy…I feel like I want like to remove it from my skin.

Similarly, participant 1 talked about both feelings in a different experience. She said,

Sometimes I wear my hijab (Head cover) and I put the bag on my shoulder, when it slides it pulls the hijab with it so I think somebody has pulled my hijab. But actually no one pulled my hijab, it's the bag. You see! But sometimes I feel like something is crawling on my hand, my face or something like that but at the end there's a reason for that…I don't think I am imagining this…

The other two students mentioned that they feel hands touching their shoulders. One participant linked her experience to the past, experiencing a real man putting a hand on her shoulder; the other participant attributed her feelings to an emotional need. Unlike the previous hallucinations, in tactile hallucinations, participants did not frame this experience as an illusion or as having any link to Jinn or Satan. In fact, they tried to frame them as real feelings that result from fears and needs.

Some of the reasons they mentioned were previous trauma, an emotional need, and the unknown but real reason. Participant 9, for example, said,

I was in a mall, and there was a person behind me who touched my shoulder and wanted to talk to me…I was afraid because I did not know him and none of my friends was there. He held my shoulder and it made me very afraid. Since then I always feel like somebody is touching my shoulder.

Another psychological reason presented was the expectations. Participant 10, for example, said,

At one point in my life, I needed my mother to be there…During the exams period in the high school she was not there, because of the COVID-19 lockdowns …so each one of us was in a different country…so I felt like she's beside me…putting her hand on me when I need her…it's not real because she's not there but I felt her with me…even when we are chatting in video calls I feel she's beside me but she's not really there.

Although participants reported that they feel these experiences very often, they were not influenced by them because they were brief. It can be argued that the repetition of these PLEs creates a kind of adaptation mechanism that is reflected in the lack of influence on their daily life and activities.

### Sensed presence hallucinations

Only three participants reported sensing the presence of somebody with high frequency. They mainly feel that somebody is watching them, and this causes them discomfort because of what other people would think of them. Participant 3, for example, said,

It causes problems…when the person does not differentiate reality from hallucinations, he is considered by people to be weird…he himself does not know how to behave or decide or even manage his life…he might put himself in abnormal situations…although it's not controlled by him but the situation causes him conflict…something inside him that he does not. Which would make think if it is natural or an illusion? So I feel it causes big problems

The immediate reaction of participants is checking the environment around them. As participant 1 said “I check cars and their numbers in case something happens”. They also framed it as a weird feeling that mainly is caused by trauma and psychological stress. As stated by participant 2,

I think it has a psychological aspect. I mean psychological trauma especially in childhood or in adolescence….traumas in these stages influence children's mental status and their stability in the future. Such traumas can lead to many hallucinations that leads them to madness, but it all the result of previous traumas in their life

Others linked this experience to watching thriller TV shows, drug addiction, and hearing jinn stories during childhood. All the above reflect trauma and external causes of these feelings, which are often framed as uncontrollable by participants.

It is important to mention that the majority of the students reported that they have never experienced a combination of two experiences. Only one participant (participant 4) reported that she felt this combination two times throughout her entire life. She told us about experiencing both auditory and tactile hallucinations. She said,

In both incidents, I experienced the same. I was sleeping and suddenly I feel somebody behind my neck …so close that I could feel them…at the same time I felt him blowing on me at the same time I heard the voice and I felt someone blowing on me and touch me.

Participants framed the condition as an abnormal one that often arises from drug addiction or mental disorder. For instance, participant 3 asserted that, “Reasons can be addiction or born disorder in the brain”.

### Delusions

Students, in this study, reported five types of delusions, and these are reference, guilt, control, grandeur, and somatic delusions. The highest occurrence was for reference, particularly thinking about messages from TV or other sources in their environment as directed to them, and the lowest (1 participant) was the grandeur.

Six participants reported experiencing special messages directed to them, mainly through social media platforms. They all reported that it does not happen regularly, but they experienced it several times in life. For example, participant 10 said,

Sometimes when I am on a social media platform, such as Instagram, there are many nice things that I see or click on, that attract me and so I feel like these are maybe messages to us when we feel bad or frustrated so like they might be messages from God… Something like this period will pass and things will get better especially if we are strong believers we tend to believe them

Three participants framed them as messages from God to help overcome feelings of sadness and frustration and one as an action of Satan and Jinn. For example, participant 2 said,

Most people believe that these messages are from God that came during times when we need it. Sometimes you have a difficult experience and all of the sudden you see a sentence in the street or TV, or picture on your mobile that looks like it is speaking to you.

Participant 12 told us about her different types of experiences where she and her friend were chatting *via* WhatsApp about the food sushi and when an advertisement for a sushi restaurant appeared first thing on her page when she logged into her Instagram account. This made her feel afraid and uncomfortable, whereby she said, “I told my friend about this and how I was afraid especially that it's the first time in my entire life an advertisement on sushi appears to me. She said that it happens to her as well.”

Five students reported feeling controlled by an unknown power. They described it as an inner voice that encouraged them to overcome hardships, perceptions, and attitudes shaped by others. They also mentioned that it made them do actions they cannot explain why they did them or who made them do these actions. They framed these feelings as real and strong feelings that happen sometimes in life. For instance, participant 9 said,

Sometimes we don't know what we are doing…we did something but when it's over you sit with yourself and say how did I do that? It's not me… I don't think that way…how could I do that? You feel somebody influenced you at that moment when you made this idea or action…Sometimes I'm overreacting…I am not myself…I do the thing without thinking about others or the harm that I may have caused…at this moment I'm out of my personality…It's an anger…For example, I hear about stuff about a certain person from other people and when I meet that person I tell them to their face you are this and that without thinking…later I say why did I talk to that person way that way? I don't even know them.

Only one participant (13) framed these thoughts scientifically. She said,

As I told you before I believe in and love physics and love evidence based things, so I believe in ghosts but not the ghosts people think about but in a scientific way…I believe they exist so when my friend comes to tell me about an impossible event even if I am realist I believe her and try to find scientific explanation and try to frame it accordingly.

All participants tend to normalize these feelings, by saying it is normal and it happens to almost everyone. However, many also assumed that the reasons can be the need to be controlled or the type of personality we have that is either easy or hard to be controlled and influenced by others. For example, participant 3 said,

Some people like this…I mean not like but they may be exposed and easy to be controlled by another person for example their ideas, actions, statements…I saw many people who became obedient to another person.

Seven students linked sudden messages and being controlled to religion. Our participants framed messages as messages from God while being controlled by invisible power as done by Jinn and Satan. Participant 10, for example, asserted,

When I was young we were supposed to begin with praying and doing everything to get closer to God…so when I see a program after program and all [programs] on praying and fasting, I feel it's a message to me that I have to be commit [to religious practices]…When we feel bad or frustrated these messages come to us …they might be from God…When we want to do something good for example to pray on time, Satan is a hidden power that tells us “no wait, postpone the prayer…why pray on time” these are the hidden powers for people in my age.

Linking delusions to religion was not present in other types of delusions such as somatic, grandeur, and guilt.

Four students reported having somatic delusions when they were asked if they ever felt that there is something weird in their bodies when others said it is not true or if they felt that one body part is bigger or smaller than usual or if there is a parasite into the body when the doctors said there is nothing. Three students reported feeling that one body part is bigger or smaller than usual. Only one reported feeling something wrong is wrong with her body, and after doing all medical checks at the hospital, there was nothing wrong. She said,

Sometimes I feel I'm not ok, something is wrong with me...when I am exhausted for several days I feel indolence… I believe I have something and I have to go to the hospital…I go to the hospital and I spend hours and they perform all tests and in the end I have nothing but I am convinced there is something wrong with me.

Participants in this study framed somatic delusions as normal and part of being a woman who wants to fit in and conform to the norms of society by having a “perfect” body. Consequently, there is a gender link to these feelings that can be referred to as a global perception of women needing to have a “perfect body”.

Three students reported having feelings of guilt. Two participants told us about their feelings of guilt toward things they did in the past. Additionally, one student reported that something happened to her sister and her sister made her feel guilty even though she does not believe she's the reason behind what happened. All three students framed these feelings as normal feelings that everyone may feel and reported them as happening very often.

For example, participant 9 said,

Yes always…self-mutilation has always worked with me, even when it comes to things that happened in the past. I tend to blame myself and say I am the reason this thing broke or that thing happened. This incident happened because of me even if the people around me were not affected by it.

Only one person reported feelings of grandeur because she believed in her “special skills” to influence people and make them succeed in their exams and life goals by talking to them and convincing them about their abilities.

Our participants reported on the different influences of the different delusions on their daily lives. When the content is positive, it affects them positively, when the content is negative, which rarely happens, it causes them discomfort but not to the extent that it affects their daily activities.

Negative reference thoughts were found to be the main type of delusions that influences them. Their reactions, in general, are to look for the source; if not found or explained, they reported on seeking refuge in God and sometimes even resorting to laughing as a coping mechanism. As participant 3 said, “I laugh with myself and I say oh God and continue in ‘*dua'* [which is what a Verbal prayer in Arabic]”.

## Discussion

PLEs are common in the general population (9, 18). In this study, PLEs were common among Arab female students enrolled at Qatar University. This provides further support for the importance of studying such a phenomenon in the Arab region, especially among women. In this study, auditory and visual hallucinations were more prevalent than olfactory and sensed presence and tactile hallucinations. Delusions were also common in our sample (six out of 12 participants), which were found to be similar to what was reported in the general population in Qatar (9).

During these interviews, religiosity and culture were clearly shaping the types and content of PLEs. For instance, in auditory hallucinations, the call for prayer (Azan) was experienced outside the actual time for prayer. Another example was, in the visual hallucinations, when one of the cultural dresses (Abbaya) was a source of PLEs. Furthermore, in the tactile hallucinations, one participant felt someone had pulled off her headscarf. This supported the previous findings that culture shapes hallucinations among clinical samples (13, 19). Kovess-Masfety et al. (19) showed that, in a sample of mainly Christian participants, those who were more religious had more experiences of PLEs. Our findings also support that, indeed even in non-clinical samples, culture and religion were shaping the type of experiences of hallucinations. It can be also argued that PLEs were among those common daily experiences that students had, including the ringing of a mobile phone and hearing a family member call out to them.

It is noteworthy that whereas reference and control delusions were highly linked to religion, grandeur and guilt were not. We can argue that this finding is related to the fact that reference and control can be easily referred to as external powers, while guilt and grandeur are internal feelings that cannot easily be linked to external powers, such as God, or other religious links to Jinn and Satan. Furthermore, we assume that, in a society where religion is intrinsic to the culture, it is expected that religious ideas are incorporated into consciousness and awareness more often.

The experiences that were described were infrequent. This was predicted as this was a non-clinical sample, and as shown in previous studies, PLEs although prevalent in non-clinical samples were infrequent (20). Thus, most of these experiences did not have an impact on daily functions or cause distress to the individuals experiencing them. Surprisingly, participants described PLEs as real even though they framed them as inner voices and illusions. Framing these experiences as real indicates the participants' attempts to deny them as delusional, because this may frame them as abnormal. So referring to them as real is another attempt to normalize these experiences. Moreover, past studies found that inner voices are thought to be precursors to full-blown auditory hallucinations that are attributed to the external space and so may be more common in the otherwise healthy population than in clinical populations. (21).

Students described PLEs as normal and caused by life events. Normalizing caused the patients to have less distress and help them adapt (2, 10). This might be one reason why students failed to describe them as negative or distressing. This study shows that auditory and visual hallucinations are being normalized more than tactile hallucinations. We assume that students tend to normalize these experiences specifically because they are highly stigmatized namely due to their link to severe mental illness, while tactile or olfactory PLEs are not typically linked to mental illness in the Arab culture. In a convenience sample of students from Qatar University, Zolezzi et al. (22) found a high prevalence of mental health stigma among students and reported that stigmatizing attitudes that were endorsed by a majority of students included believing that people with mental illness cannot have regular jobs (60.2%), that people with mental illness are dangerous (65.7%), and that they would not marry someone with a mental illness (88.9%) (P. 1,221). Their findings also suggest that cultural and religious factors influence students' beliefs about the origins and nature of the mental illness.

Religious links were common in the qualitative interviews. This was given as explanations for experiences or even shaped the type of experiences. The Arab student population in Qatar is a highly religious community, and hence, it is important to compare how similar or different these experiences were in comparison to other highly non-Muslim communities. In a qualitative comparison of clinical and non-clinical samples, the latter adopted spiritual explanations to PLEs while clinical participants (who may be paranoid and self-referential) are more likely to suggest the source as coming from other people (11). In both cases, it is linked to external powers, which differ in content and type.

The conviction that these experiences were illusions was a common theme among the participants who experienced them. They reported double-checking whether what they experienced had happened and ended up believing that they were an illusion. This may indicate an early predisposition to hallucinations in this group who have insight, whereas complete hallucinations are seen in clinical settings when insight is lost. A coherent narrative of the PLEs might serve the individual as long as it is consistent with their understanding of the world around them (23).

A lack of knowledge on why these experiences happen was common among the interviewees. Hence, we echoed previous reports such as Al-Natour et al. (24) and recommend raising awareness about these experiences, among different groups including students. We also believe that awareness workshops in high schools can be an additional tool to break the mental illness stigma in general and PLEs in particular. The educational system should aim to incorporate these workshops as part of the extracurricular activities, to encourage students to talk about mental health, and in turn to seek help when needed to mitigate any mental health issues during their university studies.

## Conclusion and limitations

The current study concludes that PLEs are relatively common among a non-clinical sample in a Middle Eastern context. Our study is an important addition to the existing literature on PLEs among non-clinical populations. Additionally, our study is one of very few similar studies in the Arab region. The main limitation of the study is the small number of participants, which in turn causes the study's limitation to indicate the need for those who experience them in non-clinical populations to seek help. We assume that the stigma surrounding mental health is one of the barriers to participate in mental health research. We assume that mental health stigma was the main barrier for men to express their willingness to participate in this study, especially since the stigma is stronger toward hallucinations and delusions. This resulted in another limitation of the study, which is having only female participants. Hence, we recommend developing and delivering awareness workshops on the importance of mental health research, as well as awareness campaigns aimed at breaking the mental health stigma. Another limitation of the study is that we did not include sensitive sociodemographic questions such as living conditions and academic performance to let the participants feel comfortable at the beginning, thus making it easier for them to talk about their PLEs openly later during the interview.

We recommend carrying out future studies that include male students as well as students from other nationalities and religions to make a strong comparison between cultures and religions, thus making the results more generalizable.

## Data availability statement

The original contributions presented in the study are included in the article/Supplementary material, further inquiries can be directed to the corresponding author/s.

## Ethics statement

The studies involving human participants were reviewed and approved by the institutional review boards of Qatar University (QU-IRB 1021-EA/19) and the Medical Research Council at Hamad Medical Corporation (MRC-03-19-032) in Qatar. Written informed consent for participation was not required for this study in accordance with the national legislation and the institutional requirements.

## Author contributions

SK and AY prepared the guidelines for the interviews. AY conducted the interviews. SK supported in transcribing the interviews. AY and SD-N analyzed the transcripts and wrote the original draft. All authors contributed to reviewing, writing the manuscript, and agreed to the final draft of the paper.

## Funding

This research was supported by the National Priorities Research Program award (NPRP-11S- 0119-180341) awarded to PW, SK, IS, and AY from the Qatar National Research Fund (a member of the Qatar Foundation).

## Conflict of interest

The authors declare that the research was conducted in the absence of any commercial or financial relationships that could be construed as a potential conflict of interest.

## Publisher's note

All claims expressed in this article are solely those of the authors and do not necessarily represent those of their affiliated organizations, or those of the publisher, the editors and the reviewers. Any product that may be evaluated in this article, or claim that may be made by its manufacturer, is not guaranteed or endorsed by the publisher.

## Author disclaimer

The statements made herein are solely the responsibility of the author.
