# Supplementary material for: Psychotic-like experiences among university female students in Qatar: A qualitative-phenomenological study
Source: Front Psychiatry. 2022 Sep 23;13:988913. doi: 10.3389/fpsyt.2022.988913 (PMC9539259; doi:10.3389/fpsyt.2022.988913)
Supplement: Supplementary file 1 [file Data_Sheet_1.PDF]

QATAR UNIVERSITY

QU-IRB

Request for Renewal of Ethics Approval For Research Involving Human Subjects

(All Forms Must be Typed)

Often filled applications forms are sent back to researchers for additional information. If care is taken to provide sufficient detail in the original application, then delays in their approval can be avoided.

All Renewals are issued for a period of one year only. Further renewals should be sought at each expiry based on the project's timeline

Project Information

QU-IRB Approval Number: **QU-IRB 1021-EA/19** Issued On: **April 29, 2019**

Expiry date of previous QU-IRB ethics approval: **28 April 2021**

Title of Research Project: **Prevalence Estimates and Potential Risk Factors of Psychotic Experiences in Student and Clinical Populations in Qatar: an Exploratory Study**

Project ID: NPRP- 11S- 0119- 180341

Principal Investigator(s): **Dr. Salma Khaled (Co-LPI), Majid Ali Al Abdulla (LPI), Arij Yehya (PI), Ali Al Sanousi (PI), Seema Patel (PI), Raed Amro (PI), Muhammad Waqar Azeem (PI), Iris Sommer (International Collaborator)**

Details pertaining to the current renewal request:

1. Please provide a brief progress report to the originally approved proposal.

The project is going as per the planned activities in the proposal; deliverables from phase one have been met according to the research plan. The first online survey component has been completed by 1200 participants. Now we are preparing for the next phase of the study, which includes conducting the qualitative study or "Cognitive interviews".

2. Is the project being carried out as described in the original submission to QU-IRB?
   x Yes ☐ No

   If no, please explain the discrepancies in the space below.

Institutional Review Board - Doha - Qatar - Hamad Medical Corporation

MRC-03-19-032 Validity: 12 07 2021 - 11 07 2022 E-stamped 14 Jul 2021

3. Has any of the research subjects suffered any serious or unexpected harms, toxicities or side effects? ☐ Yes x No

   If yes, please describe.

4. Have there been any internal or external audits of the research, preliminary (stopping rule) analyses, reports of data and safety monitoring boards.. etc? ☐ Yes x No

   If yes, please describe

5. Has there been any significant change in the <u>information</u> on which the QU-IRB provided ethics approval, e.g., new knowledge from the literature, from the present project or from other related sources? ☐ Yes x No

   If yes, please describe the new information, and indicate how it differs from that in the previous or original ethics approval, and its impact on the ethics of the research underway.

6. Have any amendments been made <u>to the application</u> since its original approval? x☐ Yes No

   If yes, please explain them.

Renewal of the IRB (find attached)

Were these amendments approved by the QU-IRB? ☐X Yes ☐ No

If yes, when? April 29, 2020 If no, why not?

Are you now requesting any amendment(s) to the original application? X Yes ☐ No

If yes, please describe and justify the proposed amendment(s).

The amendments pertains to the qualitative study, the change in Lead Principal Investigator (LPI) and the addition of new Principle Investigator (PI).

1. **<u>Change in Principal Investigators</u>**

For administration purposes, the LPI (Professor Peter Woodruff) has changed to Dr. Majid Ali Abdullah. Dr. Abdullah is the Chairman of the Psychiatry Department at Hamad Medical Cooperation. Professor Woodruff will remain fully involved in this study in the capacity of PI from his home university, University of Sheffield, UK. We would also like to add Dr. Suhad Daher-Nashif (Assistant Professor of Behavioral Sciences in College of Medicine in Qatar

MRC-03-19-032 Validity: 12 07 2021 - 11 07 2022 E-stamped 14 Jul 2021

University) as a PI to the study. She brings much needed expertise in the qualitative component of the research that we aim to conduct.

2. **Amendments on Qualitative interview for QPE Questionnaire**

The present requested amendment is in relation to modifying the interview mode for the qualitative component of this study. Due to the persisting COVID-19 pandemic, we will be using different online platforms such as Microsoft teams, Zoom, Webex, and Blackboard Ultra to conduct interviews with consenting students. We will reserve the face-to-face interviews option only to participants who prefer to have in-person interview, following all required guidelines by the QU administration in relation to the presence of students on campus.

For the recruitment criteria and study advertisement, as previously approved we will recruit Arabic speaking male and female students from different age groups (18 years or older) who may or may not have experienced psychological symptoms or diagnosed with mental illness. We will be recruiting student from Qatar University (QU). Open invitation to participate in this component of the study will be sent by email to students by the study's PIs. Students who are willing to participate in the study will approach the PIs and research team using contact information provided within the study's invitation email. Interviews will be scheduled with participants on a day and time of their preference. Preference for virtual or face-to face interview, as well as initial agreement on whether to record the interview or not record, will be decided by the eligible participants once interview is scheduled.

All audio recorded interviews, whether conducted face-to-face or through online platforms, would require subject's approval prior to the start of the interview. In face-to-face interviews, participants give their consent by signing the consent form. In virtual sessions, participants give verbal consent before the interview begins.

In this application, we have included the guidelines for and content of the cognitive interviews. The guidelines present a semi-structured interview that will allow the interviewers to assess the face validity of the questionnaire. The qualitative interview will be conducted in Arabic only. We also attached the invitation to participate and consent form within the guidelines.

7. When do you expect the project to be completed? (please provide month and year)

We expect to close recruitment for the project will within 12 months after IRB approval.

The project is expected to end in February, 2023

Important:

If the project and/or the previously approved consent form have changed since the original submission was approved, or are requested at this time, please provide the current or proposed versions and indicate where the changes were made.

Institutional Review Board - Doha - Qatar
Hamad Medical Corporation
MRC-03-19-032 Validity: 12 07 2021 - 11 07 2022 E-stamped 14 Jul 2021

Salma Khaled (Ph.D.) -Associate Professor

Social and Economic Survey Research Institute (SESRI)

T+ 974 4403 5754 (direct)

____

Principal Investigator
Address & Contacts

Date 12/1/2020

____

****

Please do not write below this line. This part is for QU-IRB use only:

Renewal Request received on: Date______________________

Renewal Issued for Period: ______________ to __________________ (One Year)

Next Renewal Due by: ______________________

جامعة قطر
QATAR UNIVERSITY
Institutional Review Board
(IRB)
APPROVED

Institutional Review Board - Doha - Qatar - Hamad Medical Corporation

MRC-03-19-032 Validity: 12 07 2021 - 11 07 2022 E-stamped 14 Jul 2021

جامعة قطر
QATAR UNIVERSITY

تعليمات للمقابلات المعرفية (الإدراكية)

التجارب الذهانية

النموذج باللغة العربية

نوفمبر، 2020

جامعة قطر
QATAR UNIVERSITY
Institutional Review Board
(IRB)
APPROVED

Institutional Review Board
Hamad Medical Corporation - Doha - Qatar
Hamad

MRC-03-19-032 Validity: 12 07 2021 - 11 07 2022 E-stamped 14 Jul 2021
